# Krill oil reduces plasma triacylglycerol level and improves related lipoprotein particle concentration, fatty acid composition and redox status in healthy young adults - a pilot study

**DOI:** 10.1186/s12944-015-0162-7

**Published:** 2015-12-15

**Authors:** Rolf K. Berge, Marie S. Ramsvik, Pavol Bohov, Asbjørn Svardal, Jan E. Nordrehaug, Espen Rostrup, Inge Bruheim, Bodil Bjørndal

**Affiliations:** Department of Clinical Science, University of Bergen, N-5020 Bergen, Norway; Department of Heart Disease, Haukeland University Hospital, N-5021 Bergen, Norway; Rimfrost AS, N-6099 Fosnavaag, Norway

**Keywords:** Krill oil, Phospholipids, EPA, DHA, DPA, Lipoprotein particle size, Choline, Carnitine, TMAO

## Abstract

**Background:**

Lipid abnormalities, enhanced inflammation and oxidative stress seem to represent a vicious circle in atherogenesis, and therapeutic options directed against these processes seems like a reasonable approach in the management of atherosclerotic disorders. Krill oil (RIMFROST Sublime®) is a phospholipid-rich oil with eicosapentaenoic acid (EPA): docosahexaenoic acid (DHA) ratio of 1.8:1. In this pilot study we determined if krill oil could favourable affect plasma lipid parameters and parameters involved in the initiation and progression of atherosclerosis.

**Methods:**

The study was conducted as a 28 days intervention study examining effect-parameters of dietary supplementation with krill oil (832.5 mg EPA and DHA per day). 17 healthy volunteers in the age group 18–36 (mean age 23 ± 4 years) participated. Plasma lipids, lipoprotein particle sizes, fatty acid composition in plasma and red blood cells (RBCs), plasma cytokines, antioxidant capacity, acylcarntines, carnitine, choline, betaine, and trimethylamine-N-oxide (TMAO) were measured before and after supplementation.

**Results:**

Plasma triacylglycerol (TAG) and large very-low density lipoprotein (VLDL) & chylomicron particle concentrations decreased after 28 days of krill oil intake. A significant reduction in the TAG/HDL cholesterol resulted. Krill oil supplementation decreased n-6/n-3 polyunsaturated fatty acids (PUFA) ratio both in plasma and RBCs. This was due to increased EPA, DHA and docosapentaenoic acid (DPA) and reduced amount of arachidonic acid (AA). The increase of n-3 fatty acids and wt % of EPA and DHA in RBC was of smaller magnitude than found in plasma. Krill oil intake increased the antioxidant capacity, double bond index (DBI) and the fatty acid anti-inflammatory index. The plasma atherogenicity index remained constant whereas the thrombogenicity index decreased. Plasma choline, betaine and the carnitine precursor, γ-butyrobetaine were increased after krill oil supplementation whereas the TMAO and carnitine concentrations remained unchanged.

**Conclusion:**

Krill oil consumption is considered health beneficial as it decreases cardiovascular disease risk parameters through effects on plasma TAGs, lipoprotein particles, fatty acid profile, redox status and possible inflammation. Noteworthy, no adverse effects on plasma levels of TMAO and carnitine were found.

## Background

Cardiovascular disease (CVD) is the leading cause of death worldwide [1]. Plasma lipids, especially cholesterol and triacylglycerol (TAG), are associated with risk for CVD and coronary artery disease [2–4]. Cholesterol is mostly transported in plasma by specific cholesterol-rich lipoproteins as low-density lipoproteins (LDL) or high-density lipoproteins (HDL). TAG is carried in very low-density lipoproteins (VLDL) and chylomicrons (CMs) Human trials have shown that plasma concentrations of high LDL-cholesterol, low HDL-cholesterol and high TAG are associated with elevated CVD risk [5, 6]. Recently, it has been found that TAG-rich lipoproteins causally influence risk for coronary artery disease (CAD) [7]. Moreover, small dense LDL particles are shown to be more atherogenic than the larger and less dense LDL particles [8]. Much research has been performed to the study of various interventions for the prevention of CVD. Since 1970, regular consumption of fatty fish or fish oils has been stated to have several positive effects on CVD because of the presence of n-3 fatty acids, namely EPA (C20:5n-3) and DHA (C22:6n-3), and lately docosapentanenoic acid (DPA, C22:5n-3) has also become a focus of interest [9, 10]. Indeed, EPA and DHA supplementations have been shown to consistently lower plasma TAG concentration [11, 12] although the efficacy of EPA and DHA in the prevention of CVD has been challenged in the recent meta-analyses [13–15]. Thus, clinical cardiovascular benefits of these n-3 fatty acids have been reported in several, but not all human trials. Most probably EPA and/or DHA do not affect CVD through a single factor, but rather due to several factors such as plasma lipid concentration [11, 12], systemic and local inflammation [16], vascular endothelial function [17, 18], and oxidative stress [17, 18].

Krill oil from Antarctic krill (*Euphausia superba*) has in the last years emerged on the market as an alternative source of EPA and DHA. An increasing number of studies with krill oil demonstrate health benefits in animals as well as in humans [19–22]. While fish oils have their bioactive n-3 fatty acids incorporated into TAG or ethylesters, krill oil has EPA and DHA esterified in phospholipids (PLs) in particular phosphatidylcholine (PC). Furthermore, the polar structure of PLs was shown to increase the bioavailability of EPA and DHA from krill oil in comparison to fish oil [23]. In addition to its high content of PLs, krill oil contains the antioxidant astaxanthin, which might enhance stability of n-3 fatty acids and preserve them from lipid oxidation.

Trimethylamine-N-oxide (TMAO) is a diet and microbiota-dependent proatherogenic metabolite and a proposed cardiovascular risk marker [24–26]. It´s precursors are betaine, choline and carnitine [24, 27], thus a high intake of PC and choline could potentially influence plasma levels of TMAO.

The main objective of this pilot study was to investigate the effect of krill oil on plasma lipids, lipoprotein particle concentrations and fatty acid profile in plasma and red blood cells (RBC). In addition, the effect of krill oil on inflammation, antioxidant capacity (AOC), and changes in plasma levels of TMAO was evaluated.

## Results

### Baseline characteristics of the study participants

The study included 18 healthy volunteers, of which all completed the supplementation period. One volunteer had a major protocol deviation, and was excluded. Thus, 17 healthy individuals (15 females, 2 males) were included in the final analysis. The mean age was 23 years (range 20–33) and the BMI 20.9 kg/m^2^ (range 17.6-25.5) (data not shown). Compliance of the intervention was >95 % for all subjects during the period (mean 100 %; range 96 % to 100 %).

### Safety parameters

No significant adverse effects or other complications were reported throughout the study. All safety parameters remained within normal limits both for males and females, and krill oil supplementation did not change these clinical parameters compared to baseline, with the exception of a small increase in gamma-glutamyltransferase (Table 1).

**Table 1:** Effect of krill oil supplementation on safety parameters

	Baseline	Endpoint	*p*-value
CRP, mg/L	1.5 ± 2.1	2.0 ± 4.3	0.6
ALT, U/L	19.4 ± 6.4	20.0 ± 8.4	0.7
AST, U/L	25.6 ± 5.4	26.6 ± 6.7	0.2
ALP, U/L	61.7 ± 18.6	61.8 ± 16.0	0.8
Bilirubin, μmol/L	6.9 ± 1.9	8.4 ± 4.9	0.2
Protein, g/L	71.5 ± 4.7	71.1 ± 4.2	0.4
Albumin, g/L	46.9 ± 2.4	46.6 ± 2.3	0.5
GT, U/L	12.9 ± 4.7	14.0 ± 5.2	0.04
Insulin, μU/mL	6.3 ± 2.5	6.6 ± 3.7	0.9
Glucose, mmol/L	4.7 ± 0.4	4.8 ± 0.4	0.1

Values are given as means ± standard deviation (*n* = 17). *P*-values ≤ 0.05 were considered statistically significant

Abbreviations: *ALT* alanine-aminotransferase, *ALP* alkaline phosphatase, *AST* asparat aminotransferase, *CRP* C-reactive protein, *GT* gamma-glutamyltransferase

### Effect on plasma lipids and lipoprotein particle size

**Fig. 1:**
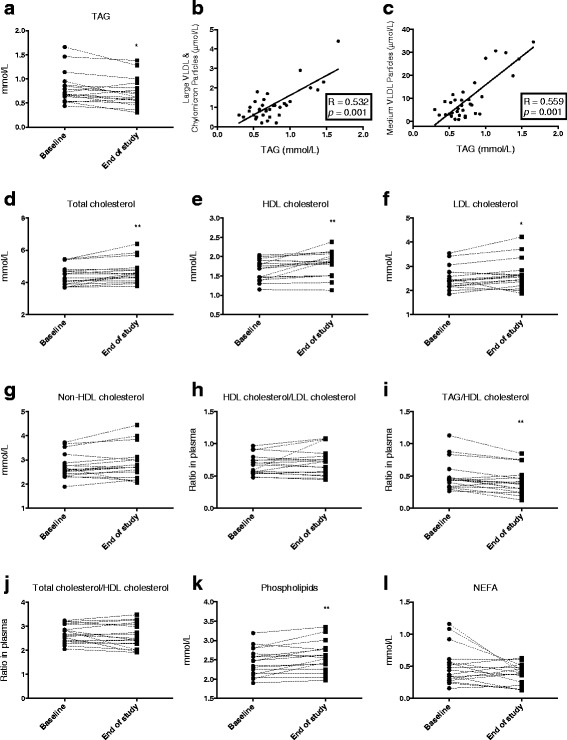
Plasma lipid profile after krill oil intake. Plasma TAG (**a**), correlation between plasma TAG and large VLDL & CM concentration (**b**), correlation between plasma TAG and medium VLDL & CM concentration (**c**), plasma total cholesterol (**d**), plasma HDL-cholesterol (**e**), plasma LDL-cholesterol (**f**), plasma non-HDL-cholesterol (**g**), plasma HDL-cholesterol/LDL-cholesterol ratio (**h**), plasma TAG/HDL-cholesterol (**i**), plasma total cholesterol/HDL-cholesterol (**j**), plasma PL (**k**), plasma NEFA (**l**). Values from all 17 participants are shown. Significant difference was determined by Wilcoxon signed-rank test (*p < 0.05, **p < 0.01). Correlation coefficients (R) were determined by Spearman correlation, and linear regressions are shown

After krill oil supplementation the plasma level of TAG decreased significantly compared to baseline (12 %, *p* = 0.016; Fig. 1a). The total VLDL & CM particle concentration tended to decrease (*p* = 0.06) and this was due to significantly decreased concentrations of large VLDL & CM particles, and partly due to medium VLDL particle concentration (*p* = 0.07; Table 2). A close positive correlation was observed between plasma TAG and large VLDL & CM and medium VLDL particles (R/rs = 0.532, p = 0.001 and R = 0.559, *p* = 0.001, respectively; Fig. 1b and c). Total plasma level of cholesterol showed a small (4 %) but significant increase by krill oil supplementation (Fig. 1d). Interestingly, the HDL-cholesterol (Fig. 1e) and LDL-cholesterol (Fig. 1f) were increased by krill oil intake whereas plasma non-HDL-cholesterol was unchanged (*p* = 0.07; Fig. 1g). The ratio of HDL-cholesterol to LDL-cholesterol remained unchanged after krill oil intake (Fig. 1h). These findings were accompanied by increased total HDL and LDL particle concentration mediated by large particle size (Table 2). Noteworthy, the small LDL particle concentration and the IDL particle concentration remained unchanged by krill oil supplementation (Table 2). Finally, the alterations in plasma lipids resulted in a significant reduction in the TAG/HDL cholesterol ratio (Fig. 1i), while the total cholesterol/HDL cholesterol ratio was unchanged (Fig. 1j). The plasma PL level increased significantly (Fig. 1k), while the plasma non-esterified fatty acid (NEFA) content remained unchanged by krill oil intake (Fig. 1l).

**Table 2:** Effect of krill oil supplementation on plasma lipoprotein concentrations

*VLDL particle concentration, nmol/L*	Baseline	End of study	*p*-value
Total VLDL & Chylomicron	32.8 ± 22.4	28.1 ± 18.8	0.06
Large VLDL & Chylomicron	1.3 ± 1.0	1.0 ± 0.6	0.04
Medium	11.1 ± 10.4	8.9 ± 8.7	0.07
Small	20.4 ± 12.9	18.3 ± 14.0	0.2
*LDL particle concentration, nmol/L*			
Total	873.6 ± 288.9	943.4 ± 352.8	0.04
Large	447.8 ± 201.5	528.5 ± 218.4	0.04
Small	352.1 ± 230.0	348.5 ± 260.8	0.7
IDL	73.7 ± 43.2	66.4 ± 76.4	0.4
*HDL particle concentration, μmol/L*			
Total	32.1 ± 5.6	33.4 ± 5.7	0.03
Large	8.7 ± 2.8	9.8 ± 3.6	0.04
Medium	10.4 ± 5.6	10.1 ± 5.7	1.0
Small	13.0 ± 3.5	13.5 ± 4.3	0.9

Values are given as means ± standard deviation (*n* = 17). *P*-values ≤ 0.05 were considered statistically significant

Abbreviations: *HDL* high-density lipoprotein, *IDL* intermediate-density lipoprotein, *LDL* low-density lipoprotein, *VLDL* very low-density lipoprotein

### Fatty acid composition in plasma and RBC

**Fig. 2:**
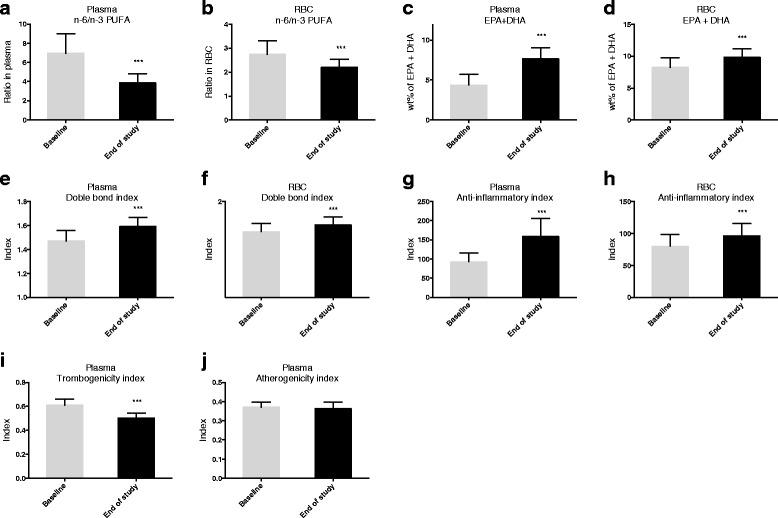
Fatty acid composition in plasma and RBC after krill oil supplementation. Plasma n-6/n-3 ratio (**a**), RBC n-6/n-3 ratio (**b**), plasma wt % of EPA + DHA (**c**), RBC wt % of EPA + DHA (omega-3 index)(**d**), plasma double bond index (**e**), RBC double bond index (**f**), plasma anti-inflammatory index (**g**), RBC anti-inflammatory index (**h**), plasma trombogenicity index (**i**), plasma atherogenicity index (**j**). Values are given as means with standard deviations (*n* = 17). Significant difference was determined by Wilcoxon signed-rank test (***p < 0.001)

When analysing the effects of krill oil on fatty acid composition in plasma and RBC, several findings were revealed. First, while the wt % of monounsaturated fatty acids (MUFAs) and n-6 fatty acids was decreased both in plasma and RBC, the wt % of polyunsaturated fatty acids (PUFAs) was increased due to increased n-3 fatty acids (Table 3). Total saturated fatty acids (SFAs) were unchanged by krill oil supplementation. Second, the increase of n-3 fatty acids was reflected by an increase of EPA (plasma, 218 fold; RBC, 100 fold), DPA (plasma, 50 fold; RBC, 10 fold) and DHA (plasma, 23 fold; RBC, 5 fold). Alpha linolenic acid (ALA, C18:3n-3) was not changed in plasma and RBC. Third, the 5 % reduction in n-6 fatty acids in plasma and RBC was primarily reflected by a decrease in the content of linoleic acid (LA, C18:2n-6) and AA (C20:4n-6; Table 3). Finally, krill oil supplementation decreased the n-6 to n-3 ratio (Fig. 2a, b) and increased wt % of EPA and DHA (often called omega-3 index in RBC), the double bond index (DBI), and the fatty acid inflammatory index in both plasma and RBC (Fig. 2c-h). Noteworthy, a more pronounced effect was observed on the n-6 to n-3 ratio and the fatty acid inflammatory index in plasma compared to RBC, despite a close correlation between wt % of EPA and DHA in plasma and RBC (R =0.665, *p* = 0.0001; data not shown). It is reported that the EPA level in fish oil is responsible for lowering plasma TAG in animals [28–30]. However, neither plasma EPA and DHA alone correlated with plasma TAG and VLDL & CM particle concentration (data not shown). Moreover, no associations between the wt % of EPA and DHA in plasma and RBC were found with plasma TAG. It was of interest, however, to observe that krill oil supplementation decreased the plasma thrombogenicity index (Fig. 2i) whereas the atherogenicity index was unchanged (Fig. 2j).

**Table 3:** Effect of krill oil on plasma and red blood cell (RBC) fatty acid profile

	Plasma			RBC		
Fatty acids Wt %	Baseline	End of study	*p*-value	Baseline	End of study	*p*-value
SFA	31.8 ± 0.7	31.6 ± 1.0	0.2	40.9 ± 0.6	40.7 ± 0.6	0.8
MUFA	25.2 ± 2.3	23.6 ± 2.9	0.007	17.5 ± 0.7	17.3 ± 0.9	0.02
PUFA	42.8 ± 2.7	44.6 ± 3.2	0.005	41.4 ± 0.7	41.8 ± 0.8	0.003
*n-6 PUFAs*	37.0 ± 2.7	35.1 ± 3.7	0.008	29.9 ± 1.8	28.5 ± 1.7	<0.0001
C18:2n-6 (LA)	27.7 ± 2.7	26.8 ± 3.5	0.7	9.1 ± 1.0	8.7 ± 1.0	0.02
C20:3n-6	1.5 ± 0.3	1.3 ± 0.3	0.0008	1.5 ± 0.4	1.4 ± 0.3	0.002
C20:4n-6 (AA)	7.0 ± 1.6	6.3 ± 1.4	0.0007	15.9 ± 1.5	15.1 ± 1.4	<0.0001
C22:5n-6	0.1 ± 0.03	0.1 ± 0.02	<0.0001	0.4 ± 0.1	0.4 ± 0.07	<0.0001
*n-3 PUFAs*	5.7 ± 1.5	9.4 ± 1.6	<0.0001	11.3 ± 1.7	13.2 ± 1.5	<0.0001
C18:3n-3 (ALA)	0.7 ± 0.2	0.7 ± 0.3	0.8	0.2 ± 0.03	0.2 ± 0.04	0.8
C20:5n-3 (EPA)	1.1 ± 0.6	3.6 ± 0.8	0.0002	1.2 ± 0.4	2.5 ± 0.4	<0.0001
C22:5n-3 (DPA)	0.6 ± 0.1	0.8 ± 0.2	<0.0001	2.8 ± 0.4	3.1 ± 0.3	<0.0001
C22:6n-3 (DHA)	3.2 ± 0.8	3.9 ± 0.7	0.0002	7.0 ± 1.2	7.4 ± 1.0	<0.0001

Values indicate plasma and RBC content by percent weight (wt %)

*P*-values ≤ 0.05 were considered statistically significant

Abbreviations: *AA* arachidonic acid, *ALA* alpha linolenic acid, *DHA* docosahexaenoic acid, *DPA* docosapentaenoic acid, *EPA* eicosapentaenoic acid, *LA* linoleic acid, *MUFAs* monounsaturated fatty acids, *n-3* omega-3, *n-6* omega-6, *PUFAs* polyunsaturated fatty acids, *SFAs* saturated fatty acids

### Effect on antioxidant status

**Fig. 3:**
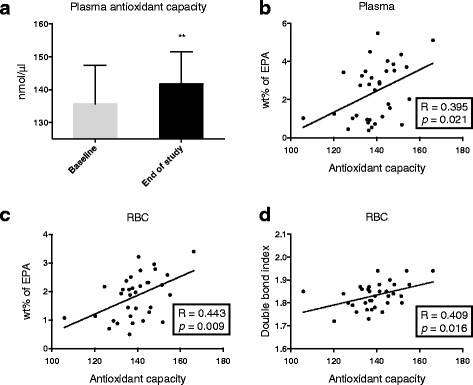
Antioxidant capacity (AOC) status after krill oil intake. Plasma level of AOC (**a**), correlation between plasma EPA concentration and AOC (**b**), correlation between EPA concentration in RBC and AOC (**c**), correlation between DBI and AOC in RBC (**d**). AOC is given as mean with standard deviation (*n* = 14). Significant difference was determined by Wilcoxon signed-rank test (**p < 0.01). Correlation coefficients (R) were determined by Spearman correlation, and linear regressions are shown

Oxidative stress is associated with decreased fatty acid unsaturation and increased lipid peroxidation [31]. The plasma total antioxidant capacity (AOC) significantly increased after krill oil intake (Fig. 3a) and it was of interest that AOC positively correlated to plasma EPA concentration (R = 0.395, *p* = 0.021; Fig. 3b), RBC EPA concentration (R = 0.443, *p* = 0.009: Fig. 3c) and RBC DBI (R = 0.409, *p* = 0.016; Fig. 3d). No correlation was found between AOC and DHA concentrations in plasma and RBC after krill oil intake, but the wt % of EPA and DHA both in plasma and RBC correlated to AOC (data not shown).

### Effect on systemic inflammation

Krill oil supplementation in humans could be expected to have anti-inflammatory potential in plasma as the fatty acid anti-inflammatory index increased (Fig. 2g). However, the plasma cytokines IL-1β, IL-10, IL-17, granulocyte colony-stimulating factor (G- CSF) and tumor necrosis factor alpha (TNFα) were unchanged after krill oil intake (data not shown).

### Effect on choline, betaine, carnitine and TMAO

**Fig. 4:**
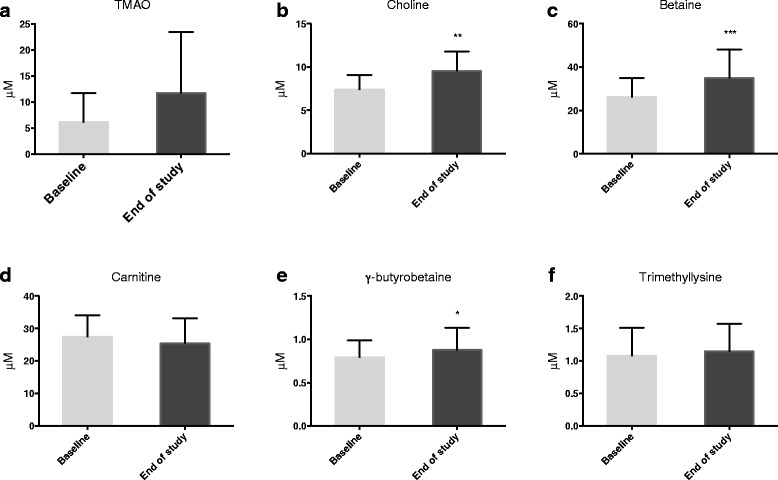
Plasma TMAO, carnitine, and carnitine precursors after krill oil intake. Plasma levels of TMAO (**a**), choline (**b**), betaine (**c**), carnitine (**d**), γ-butyrobetaine (**e**), and trimethyllysine (**f**). Values are given as means with standard deviation (*n* = 17). Significant difference was determined by Wilcoxon signed-rank test (*p < 0.05, **p < 0.01, ***p < 0.001)

TMAO is a substance formed in the liver from trimethylamine generated by the gut microbiota from dietary PC, choline, betaine and carnitine [24–27]. Krill contains both PC and TMAO, while krill oil only contains PC. The plasma level of TMAO was not affected by krill oil intake (Fig. 4a). Both choline and its metabolite betaine increased in plasma after 28 days with krill oil supplementation (Fig. 4b, c), whereas the plasma carnitine level remained constant (Fig. 4d). The carnitine precursor, γ-butyrobetaine was significantly increased in plasma by krill oil intake compared to baseline values (Fig. 4e). The other carnitine precursor, trimethyllysine, remained unchanged over the intervention period (Fig. 4f).

### Effect on plasma acylcarnitine level

**Fig. 5:**
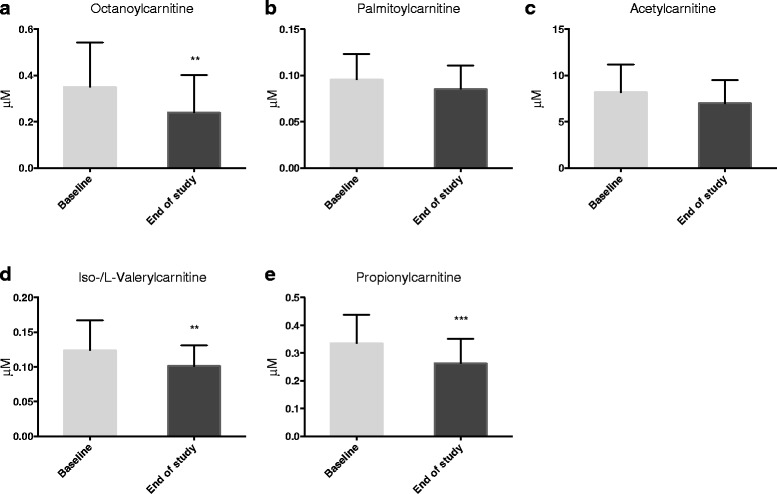
Plasma acylcarnitines after krill oil intake. Plasma levels of octanoylcarnitine (**a**), palmitoylcarnitine (**b**), acetylcarnitine (**c**), iso-/L-valerylcarnitine (**d**), and propionylcarnitine (**e**). Values are given as means with standard deviation (*n* = 17). Significant difference was determined by Wilcoxon signed-rank test (**p < 0.01, ***p < 0.001)

It is reported that high serum concentrations of long-and medium plasma acylcarnitines are linked to increased risk of disease progression with CAD, especially in heart failure patients [32]. Moreover, increased plasma concentrations of these acylcarnitines may indicate defects in mitochondria function [33]. In the present study, plasma octanoylcarnitine level was decreased after krill oil intake (Fig. 5a), whereas the palmitoylcarnitine and acetylcarnitine levels remained unchanged (Fig. 5b, c). In the current study a 15 to 20% (compared to baseline) reduction resulted for the short-chain acylcarnitines, namely iso-/L-valerylcarnitine and propionylcarnitine (Fig. 5d, e).

## Discussion

The present study has shown that 28 days supplementation of krill oil had significant biological effects in healthy subjects. Moreover, intake of this EPA-and DHA rich PC-oil beneficially affected several parameters linked to CVD-risk, including plasma lipids, lipoprotein particle concentrations, fatty acid composition, redox status and potentially inflammation and thrombogenicity. However, a small increase in cholesterol was observed. The krill oil intake did not change the safety parameters (Table 1).

Krill oil intake reduced plasma TAG levels due to decreased concentration of large VLDL & CM particles (Table 2), concomitant with increased concentration of large HDL particles and decreased TAG/HDL ratio (Fig. 1i). Large LDL particles also increased after 28 days krill oil supplementation and this resulted in a modest, but significantly higher plasma total cholesterol level (Fig.1d). However, total cholesterol/HDL, a robust risk parameter in European populations [34], was unaltered (Fig. 1j). The increase in large HDL particles in response to n-3 PUFA supplementation has been demonstrated in several studies [35, 36], and may be explained by a reduced exposure to cholesterol ester transfer protein (CETP)-mediated transfer of cholesterol from HDL particles due to a lower concentration of TAG rich large VLDL and CM. Interestingly, the increased concentration of large HDL-and LDL particles has mainly been found with DHA, but not EPA supplementation [37, 38]. Noteworthy, the concentration of the more atherogenic small LDL particles remained constant after krill oil supplementation.

It has been assumed that EPA or DHA, or both, are responsible for the TAG-lowering effect after supplementation of n-3 PUFA. Most studies have used fish oil with various contents of EPA and DHA, and only a few studies have examined the effect of EPA and DHA separately to determine for the effects for each fatty acid. Previous studies have demonstrated n-3 PUFA supplementation to lower plasma TAG levels when ingesting 2–4 g EPA and DHA per day for 6 weeks or longer, although the effect seem to be strongest among people with an unfavourable risk profile [11, 12, 39]. Supplementation with 4 g EPA per day for 6–7 weeks has been shown to reduce TAG by 21 % [40] and 23 % [38] in mildly hyperlipidaemic subjects, and by 12 % in healthy subjects [41]. The present study showed a reduction in TAG levels (12 %, *p* = 0.02) in healthy young adults after 28 days of supplementation with 832.5 mg EPA and DHA from krill oil with an EPA:DHA ratio of approximately 1.8:1. This indicates a high bioavailability of krill oil (RIMFROST Sublime®) and a subsequent rapid effect on lipid metabolism. Moreover, this is in line with a recent study in adults with high TAG levels where a dose of 0.5-2 g/day krill oil significantly reduced plasma TAG [20].

In humans, supplementation of EPA gives rise to EPA and DPA, but not DHA whereas DHA supplementation gives formation of DPA and EPA [41, 42]. In keeping with these findings our present study gave rise in DPA both in RBC (approximately 10 %) and plasma (approximately 23 %) and was greater than would be expected, as krill oil does not contain DPA (Table 4). Previous studies have shown similar results, both in RBC [43] and in plasma [21, 44, 45]. Moreover, in the present study the increase in DPA both in plasma and RBCs was higher than DHA. We have recently found that PLs from herring roe with a DHA:EPA ratio of about 3:1 decreased plasma TAG in healthy subjects [46]. Despite a dose of 132 mg DPA/day and the possibility of formation of DPA from EPA, DPA levels were not influenced by herring roe PLs. This suggests that DPA is less important in mediating TAG lowering effects both in krill oil and oil from herring.

**Table 4:** Composition of the krill oil (wt %)

Fatty acid^a^	Krill oil capsules
∑ SFAs	36.5
C14:0	11.4
C16:0	24.0
C18:0	1.3
C20:0	<0.1
C22:0	<0.1
∑ MUFAs	26.2
C16:1*n-*7	5.5
C18:1 (*n-*9) + (n-7) + (n-5)	19.3
C20:1 (*n-*9) + (n-7)	1.0
C22:1 (*n-*11) + (*n-*9) + (n-7)	0.6
C24:1*n-*9	<0.1
*n-*6 PUFAs	2.6
C18:2*n-*6 (LA)	2.1
C18:3*n-*6	0.2
C20:2*n-*6	0.2
C20:3*n-*6	<0.1
C20:4*n-*6 (AA)	0.2
C22:4*n-*6	<0.1
*n-*3 PUFAs	34.1
C18:3*n-*3 (ALA)	1.1
C18:4*n-*3	3.7
C20:3*n-*3	<0.1
C20:4*n-*3	0.3
C20:5*n-*3 (EPA)	18.0
C21:5*n-*3	0.5
C22:5*n-*3 (DPA)	0.5
C22:6*n-*3 (DHA)	10.1
Lipids^b^	
* Total polar lipids*	*47.6*
Phosphatidylcholin (PC)	44.0
Lyso-PC	2.3
Phosphatidylethanolamine (PE)	1.4
Free fatty acids	3.1
* Total neutral lipids*	*52.4*
Triacylglycerol (TAG)	44.0
Diacylglycerol	2.1
Monoacylglycerol	<0.1
Free fatty acids	3.1

^a^g/100 g extracted fatty acids. ^b^g/100 g extracted lipids

Abbreviations: see legend to Table 3

Studies in cultured rat hepatocytes have shown that EPA increases mitochondrial fatty acid oxidation [30], and as recently reviewed, plasma acylcarnitine levels can give an indication on mitochondrial function [33]. When acyl-CoAs accumulate in the mitochondrial matrix, acylcarnitine formation is favoured, which can exit the mitochondrion and access the peripheral circulation. The intra-mitochondrial relationship between acyl-CoA and free CoA is reflected by the extra-mitochondrial acylcarnitine to free carnitine ratio, given that any mitochondrial acyl-CoA accumulation leads to a corresponding increase in acylcarnitines. In situations of inefficient β-oxidation, intermediates such as octanoyl- and palmitoylcarnitine will be formed, which are considered toxic [47]. Elevated palmitoylcarnitine has been associated with poor prognosis in chronic heart failure (CHF) patients [32]. In the present study the plasma level of octanoylcarnitine was decreased by krill oil supplementation. Altogether, this suggests that the krill oil intervention may be responsible for more efficient fatty acid oxidation of medium-chain length fatty acids. Noteworthy, this is in consistence with findings in animal studies where krill oil was shown to influence metabolic pathways, including fatty acid oxidation, lipid biosynthesis and acylcarntine turnover [48, 49].

Choline is mainly utilized for PC biosynthesis, which is required for membrane formation and can take place through two pathways, namely CDP-choline (Kennedy) pathway and the phospatidylethanolamine N-methyltransferase (PEMT) pathway. Moreover, choline can be oxidized to betaine, which is involved in the remethylation of homocysteine to methionine in the one-carbon cycle [50]. In the present study plasma choline and betaine was increased by krill supplementation, indicating an increased PC degradation and increased choline oxidation, respectively. Choline is an important nutrient and is predominantly obtained from the diet [51, 52], although it can be synthesised *de novo.* In a recent European study, the average choline intake was below the adequate intake (set by the Institute of Medicine in the USA) in most of the population groups considered [53]. Thus, krill oil can be a well-suited supplement for populations vulnerable to choline deficiency.

TMAO is formed in the liver from trimethylamine, a product generated by the gut microbiota from dietary PC, choline and carnitine. Trimethylamine can also be generated from betaine [26]. Recent studies have shown that TMAO is a diet and microbiota-dependent proatherogenic metabolite and cardiovascular risk marker [24–27]. Interestingly, in the present study the plasma TMAO level remained constant by krill oil supplementation, demonstrating that the extra choline intake did not influence TMAO generation.

AA is a precursor for prostaglandins [16] and is considered proinflammatory. Krill oil supplementation decreased the level of AA and increased amounts of n-3 PUFAs in plasma and RBC resulting in an increased anti-inflammatory fatty acid index. This implies that krill oil intake has anti-inflammatory properties. The plasma cytokines, however, remained unchanged. The plasma total antioxidant status significantly increased after krill oil supplementation (Fig. 3a) and it was of interest that AOC positively correlated with the plasma and RBC levels of EPA and wt % of EPA and DHA. Moreover, plasma AOC correlated with RBC DBI but no correlation was found between AOC and DHA concentrations in plasma and RBC after krill oil intake (data not shown). Whether the presence of astaxanthin is responsible for the oxidative protective effect observed, should be considered [54]. This is an important point, as the removal of protective trace compounds in the generation of highly purified PUFAs has been reported to reduce the beneficial effect of fish oil supplements [55].

## Conclusions

28 days of krill oil supplementation in healthy young adults reduced plasma TAG and large VLDL & CM particle concentration, whereas the concentrations of large HDL and LDL were increased. The changes in fatty acid composition in plasma and RBC were accompanied by increased n-3 PUFAs, decreased level of AA and increased the plasma antioxidant status. Furthermore, no adverse effects of krill oil intake on plasma TMAO levels were found. Thus, a dose of 832.5 mg EPA and DHA from krill oil had beneficial biological effects in persons with a normal lipid profile, and may prevent the development of several risk factors related to CVD.

## Methods

### Study population

18 subjects in the age group 18–36, with a body mass index (BMI; in kg/m^2^) of 17–29.9 and in a generally good health, were recruited to the pilot study. Exclusion criteria were disease related to fat metabolism or digestion, acute or chronic inflammatory conditions, liver dysfunctions, pregnancy (all self-reported), obesity (defined as a BMI ≥30), inability to swallow capsules and unwillingness to take blood samples.

### Intervention

The intervention used was krill oil capsules (RIMFROST Sublime ®, Rimfrost AS, Fosnavaag, Norway). The lipid- and fatty acid composition of the oil is presented in Table 4. Each capsule (500 mg) contained 210 mg PLs and 120 mg n-3 PUFA, of which 60 mg was EPA and 32.5 mg DHA. The participants received 9 capsules per day for 28 days, corresponding to a daily dose of 832.5 mg EPA and DHA Moreover, the oil contained 192 mg astaxanthin per kg.

### Study procedures

Subjects were recruited through advertisements presented at the University of Bergen and the Bergen University College (Norway). After written informed consent to participate was provided, blood samples were drawn, and body weight and height were recorded to calculate body mass index (BMI; kg/m^2^). Study participants were interviewed about their dietary intake of food stuffs rich in n-3 fatty acids and PLs, focusing on the amount and frequency of the last month intake. Participants were advised to maintain a steady intake of these food items during the study period, refrain from the use of all nutritional supplements, and to maintain their body weight. At the end of the study, all participants were asked to answer questionnaires to assess self-reported compliance and product tolerance. The 18 subjects included all completed the intervention period. However, one subject (male) was excluded from the final analysis due to protocol deviation revealed by the questionnaires.

### Blood sample collection

Blood samples were collected from subjects fasted overnight and seated for 15 minutes, at the beginning (baseline) and end of the study. At both time points, blood samples were centrifuged and EDTA-plasma and serum was collected after a minimum of 15 minutes and maximum of 30 minutes at room temperature. Blood samples for isolation of RBC was drawn in EDTA-tubes, centrifuged at 3000 rpm for 10 minutes, and plasma and interface removed. RBC was then washed three times in phosphate buffered saline (PBS), with subsequent centrifugation and removal of the buffy coat (white blood cells) between each wash. Finally, the original volume was reconstituted using PBS. All samples were aliquoted and stored at −80°C for further analysis.

### Total fatty acid composition in the red blood cells (RBC) and in plasma

The total fatty acid composition was analysed in RBC and in EDTA-plasma, as previously described [56].

The omega-3 index is defined as the sum of EPA and DHA in RBC, expressed as a percentage of the total fatty acid (TFA) content. The anti-inflammatory index is defined as the sum of EPA (C20:5n-3), dihomo-gamma-linolenic acid (DGLA, C20:3n-6), DHA (C22:6n-3) and DPA (C22:5n-3), divided on AA (C20:4n-6), multiplied with 100. The double bound index (DBI) is defined as (MUFA + 2*(C18:2n6 + C20:2n6 + C22:2n6) + 3*(C18:3n6 + C18:3n3 + C20:3n9 + C20:3n6) + 4*(C18:4n3 + C20:4n6 + C20:4n3 + C22:4n6) + 5*(C20:5n3 + C21:5n3 + C22:5n6 + C22:5n3) + 6*(C22:6n3))/TFA. The atherogenicity index (AI) and the thrombogenenicity (TI) were determined according to Ulbricht and Southgate [57] equations: AI = (C12:0 + 4*C14:0 + C16:0)/(ΣMUFA + Σ(n-6 PUFA) + Σ(n-3 PUFA)). TI = (C14:0 + C16:0 + C18:0)/(0.5*ΣMUFA + 0.5*Σ(n-6 PUFA) + 3*Σ(n-3 PUFA) + (Σ(n-3 PUFA)/Σ(n-6 PUFA)), where ΣMUFA, Σ(n-6 PUFA), and Σ(n-3 PUFA) are the sum of MUFA, n-6 and n-3 PUFA in wt % of total fatty acids, respectively.

### Lipids and lipoproteins

Lipids were measured enzymatically in EDTA-plasma on a Hitachi 917 system (Roche Diagnostics GmbH, Mannheim, Germany) using the triacylglycerol (GPO-PAP), cholesterol (CHOD-PAP), HDL-cholesterol plus and LDL-cholesterol plus kit from Roche Diagnostics, the non-esterified fatty acid (NEFA FS) kit and the Phospholipids FS kit 17 from DiaSys Diagnostic Systems GmbH (Holzheim, Germany).

Lipoprotein size was measured in EDTA-plasma with nuclear magnetic resonance (NMR) spectroscopy by LabCorp Inc (Burlington, NC, USA), using the *NMR LipoProfile*® test (http://www.labcorp.com).

### Biochemical analysis

Plasma choline, betaine, trimethyllysine (TML), butyrobetaine and trimethylamine N-oxide (TMAO), as well as L-carnitine, acetylcarnitine, proponylcarnitine, octanoylcarnitine, iso-/L-valerylcarnitine and palmitoylcarnitine, were analysed in EDTA-plasma using LC/MS/MS as previously described [58, 59].

Clinical laboratory measurements, including safety parameters were measured in EDTA-plasma using routine methods at the central laboratory at Haukeland University Hospital (Bergen, Norway).

Total AOC of plasma was measured using the total antioxidant capacity kit (Abcam, Cambridge, UK) according to the manufacturer’s instructions. The protein mask was not used, enabling the analysis of both small molecule antioxidants and proteins ability to reduce Cu^2+^ to Cu^+^. In brief, EDTA-plasma was allowed to reduce Cu^2+^ for 1.5 hour at room temperature on an orbital shaker. The absorbance was measured at 570 nm using a plate reader. Results were expressed as trolox equivalents according to a trolox standard curve.

The levels of IL-1β, IL-10, IL-17, G-CSF and TNF-α were measured in plasma samples using a custom-made multiplex MILLIPLEX MAP kit (Millipore Corp., St. Charles, IL, USA), and the assay solution was read by the Bio-Plex array reader (Bio-Rad, Hercules, CA, USA) and determined with the Bio-Plex Manager Software 4.1.

### Questionnaires

All participants were asked to answer a questionnaire to assess compliance, supplementation regime, product tolerance and to report of possible gastrointestinal (GI) symptoms occurring when ingesting the supplement. Compliance was further determined by capsule logs and validated by count of capsules in surplus. An authorised clinical nutritionist interviewed the participants into the regime, and general product tolerance.

### Ethical aspects

All examinations and blood samples were performed at the Department of Clinical Science, University of Bergen, Norway. The study protocol was approved by the Regional Ethics Committee of Northern Norway (Registration number 2013/2152), and registered on ClinicalTrials.gov (NCT02053415). All procedures were carried out in accordance with the ethical standards of the Helsinki declaration of 1975, revised in 2004, for experiments involving humans (http://www.wma.net/en/30publications/10policies/b3/index.html).

### Statistics

Data sets were analysed using Prism Software (Prism Graph-Pad Software, version 6, San Diego, California, USA) to determine statistical significance. The results are reported as means per group with their standard deviations (SD), with exception of age, BMI and compliance, which is reported as mean with range. Wilcoxon signed-rank test was performed to evaluate statistical differences of matched pairs. Correlation coefficients (R) were determined by Spearman correlation, and linear regressions are shown in selected figures. P-values < 0.05 were considered significant.

## References

[1] The top 10 causes of death - Fact sheet. http://www.who.int/mediacentre/factsheets/fs310/en. Accessed 4 Dec 2015]

[2] World Health Organization (WHO) (2007). Prevention of cardiovascular disease: guidelines for assessment and management of total cardiovascular risk.

[3] Emberson JR, Whincup PH, Morris RW, Walker M (2003). Re-assessing the contribution of serum total cholesterol, blood pressure and cigarette smoking to the aetiology of coronary heart disease: impact of regression dilution bias. Eur Heart J.

[4] Yusuf S, Hawken S, Ounpuu S, Dans T, Avezum A, Lanas F (2004). Effects of potentially modifiable risk factors associated with myocardial infarction in the 52 countries (the INTERHEART study): case–control study. Lancet.

[5] Barter P, Gotto AM, LaRosa JC, Maroni J, Szarek M, Grundy SM (2007). HDL cholesterol, very low levels of LDL cholesterol, and cardiovascular events. N Engl J Med.

[6] Miller M, Cannon CP, Murphy SA, Qin J, Ray KK, Braunwald E (2008). Impact of triglyceride levels beyond low-density lipoprotein cholesterol after acute coronary syndrome in the PROVE IT-TIMI 22 trial. J Am Coll Cardiol.

[7] Egeland GM, Igland J, Sulo G, Nygard O, Ebbing M, Tell GS (2015). Non-fasting triglycerides predict incident acute myocardial infarction among those with favourable HDL-cholesterol: Cohort Norway. Eur J Prev Cardiol.

[8] Galeano NF, Al-Haideri M, Keyserman F, Rumsey SC, Deckelbaum RJ (1998). Small dense low density lipoprotein has increased affinity for LDL receptor-independent cell surface binding sites: a potential mechanism for increased atherogenicity. J Lipid Res.

[9] Bucher HC, Hengstler P, Schindler C, Meier G (2002). N-3 polyunsaturated fatty acids in coronary heart disease: a meta-analysis of randomized controlled trials. Am J Med.

[10] Delgado-Lista J, Perez-Martinez P, Lopez-Miranda J, Perez-Jimenez F (2012). Long chain omega-3 fatty acids and cardiovascular disease: a systematic review. Br J Nutr.

[11] Mori TA, Woodman RJ (2006). The independent effects of eicosapentaenoic acid and docosahexaenoic acid on cardiovascular risk factors in humans. Curr Opin Clin Nutr Metab Care.

[12] von Schacky C (2006). A review of omega-3 ethyl esters for cardiovascular prevention and treatment of increased blood triglyceride levels. Vasc Health Risk Manag.

[13] Enns JE, Yeganeh A, Zarychanski R, Abou-Setta AM, Friesen C, Zahradka P (2014). The impact of omega-3 polyunsaturated fatty acid supplementation on the incidence of cardiovascular events and complications in peripheral arterial disease: a systematic review and meta-analysis. BMC Cardiovasc Disord.

[14] Kotwal S, Jun M, Sullivan D, Perkovic V, Neal B (2012). Omega 3 Fatty acids and cardiovascular outcomes: systematic review and meta-analysis. Circ Cardiovasc Qual Outcomes.

[15] Rizos EC, Ntzani EE, Bika E, Kostapanos MS, Elisaf MS (2012). Association between omega-3 fatty acid supplementation and risk of major cardiovascular disease events: a systematic review and meta-analysis. Jama.

[16] Calder PC (2006). n-3 polyunsaturated fatty acids, inflammation, and inflammatory diseases. Am J Clin Nutr.

[17] Mori TA, Beilin LJ (2004). Omega-3 fatty acids and inflammation. Curr Atheroscler Rep.

[18] Massaro M, Scoditti E, Carluccio MA, De Caterina R (2008). Basic mechanisms behind the effects of n-3 fatty acids on cardiovascular disease. Prostaglandins Leukot Essent Fatty Acids.

[19] Berge K, Musa-Veloso K, Harwood M, Hoem N, Burri L (2014). Krill oil supplementation lowers serum triglycerides without increasing low-density lipoprotein cholesterol in adults with borderline high or high triglyceride levels. Nutr Res.

[20] Burri L, Johnsen L (2015). Krill products: an overview of animal studies. Nutrients.

[21] Ulven SM, Kirkhus B, Lamglait A, Basu S, Elind E, Haider T (2011). Metabolic effects of krill oil are essentially similar to those of fish oil but at lower dose of EPA and DHA, in healthy volunteers. Lipids.

[22] Tillander V, Bjorndal B, Burri L, Bohov P, Skorve J, Berge RK (2014). Fish oil and krill oil supplementations differentially regulate lipid catabolic and synthetic pathways in mice. Nutr Metab (Lond).

[23] Kohler A, Sarkkinen E, Tapola N, Niskanen T, Bruheim I (2015). Bioavailability of fatty acids from krill oil, krill meal and fish oil in healthy subjects--a randomized, single-dose, cross-over trial. Lipids Health Dis.

[24] Koeth RA, Wang Z, Levison BS, Buffa JA, Org E, Sheehy BT (2013). Intestinal microbiota metabolism of L-carnitine, a nutrient in red meat, promotes atherosclerosis. Nat Med.

[25] Tang WH, Wang Z, Levison BS, Koeth RA, Britt EB, Fu X (2013). Intestinal microbial metabolism of phosphatidylcholine and cardiovascular risk. N Engl J Med.

[26] Wang Z, Klipfell E, Bennett BJ, Koeth R, Levison BS, Dugar B (2011). Gut flora metabolism of phosphatidylcholine promotes cardiovascular disease. Nature.

[27] Rebouche CJ, Seim H (1998). Carnitine metabolism and its regulation in microorganisms and mammals. Annu Rev Nutr.

[28] Ikeda I, Wakamatsu K, Inayoshi A, Imaizumi K, Sugano M, Yazawa K (1994). alpha-Linolenic, eicosapentaenoic and docosahexaenoic acids affect lipid metabolism differently in rats. J Nutr.

[29] Willumsen N, Hexeberg S, Skorve J, Lundquist M, Berge RK (1993). Docosahexaenoic acid shows no triglyceride-lowering effects but increases the peroxisomal fatty acid oxidation in liver of rats. J Lipid Res.

[30] Willumsen N, Skorve J, Hexeberg S, Rustan AC, Berge RK (1993). The hypotriglyceridemic effect of eicosapentaenoic acid in rats is reflected in increased mitochondrial fatty acid oxidation followed by diminished lipogenesis. Lipids.

[31] Assies J, Mocking RJ, Lok A, Ruhe HG, Pouwer F, Schene AH (2014). Effects of oxidative stress on fatty acid- and one-carbon-metabolism in psychiatric and cardiovascular disease comorbidity. Acta Psychiatr Scand.

[32] Ueland T, Svardal A, Oie E, Askevold ET, Nymoen SH, Bjorndal B (2013). Disturbed carnitine regulation in chronic heart failure--increased plasma levels of palmitoyl-carnitine are associated with poor prognosis. Int J Cardiol.

[33] Reuter SE, Evans AM (2012). Carnitine and acylcarnitines: pharmacokinetic, pharmacological and clinical aspects. Clin Pharmacokinet.

[34] McQueen MJ, Hawken S, Wang X, Ounpuu S, Sniderman A, Probstfield J (2008). Lipids, lipoproteins, and apolipoproteins as risk markers of myocardial infarction in 52 countries (the INTERHEART study): a case–control study. Lancet.

[35] Calabresi L, Villa B, Canavesi M, Sirtori CR, James RW, Bernini F (2004). An omega-3 polyunsaturated fatty acid concentrate increases plasma high-density lipoprotein 2 cholesterol and paraoxonase levels in patients with familial combined hyperlipidemia. Metabolism.

[36] Maki KC, Lawless AL, Kelley KM, Dicklin MR, Kaden VN, Schild AL (2011). Effects of prescription omega-3-acid ethyl esters on fasting lipid profile in subjects with primary hypercholesterolemia. J Cardiovasc Pharmacol.

[37] Kelley DS, Siegel D, Vemuri M, Mackey BE (2007). Docosahexaenoic acid supplementation improves fasting and postprandial lipid profiles in hypertriglyceridemic men. Am J Clin Nutr.

[38] Neff LM, Culiner J, Cunningham-Rundles S, Seidman C, Meehan D, Maturi J (2011). Algal docosahexaenoic acid affects plasma lipoprotein particle size distribution in overweight and obese adults. J Nutr.

[39] European Food Safety Authority (EFSA) Panel on Dietetic Products NaAN (2010). Scientific Opinion on the substantiation of health claims related to eicosapentaenoic acid (EPA), docosahexaenoic acid (DHA), docosapentaenoic acid (DPA). Book Scientific Opinion on the substantiation of health claims related to eicosapentaenoic acid (EPA), docosahexaenoic acid (DHA), docosapentaenoic acid (DPA).

[40] Nestel P, Shige H, Pomeroy S, Cehun M, Abbey M, Raederstorff D (2002). The n-3 fatty acids eicosapentaenoic acid and docosahexaenoic acid increase systemic arterial compliance in humans. Am J Clin Nutr.

[41] Grimsgaard S, Bonaa KH, Hansen JB, Nordoy A (1997). Highly purified eicosapentaenoic acid and docosahexaenoic acid in humans have similar triacylglycerol-lowering effects but divergent effects on serum fatty acids. Am J Clin Nutr.

[42] Hansen J-B, Grimsgaard S, Nilsen H, Nordøy A, Bønaa KH (1998). Effects of highly purified eicosapentaenoic acid and docosahexaenoic acid on fatty acid absorption, incorporation into serum phospholipids and postprandial triglyceridemia. Lipids.

[43] Katan MB, Deslypere JP, van Birgelen AP, Penders M, Zegwaard M (1997). Kinetics of the incorporation of dietary fatty acids into serum cholesteryl esters, erythrocyte membranes, and adipose tissue: an 18-month controlled study. J Lipid Res.

[44] Ramprasath VR, Eyal I, Zchut S, Jones PJ (2013). Enhanced increase of omega-3 index in healthy individuals with response to 4-week n-3 fatty acid supplementation from krill oil versus fish oil. Lipids Health Dis.

[45] Von Schacky C, Fischer J, Weber PC (1985). Long- term effects of dietary marine omega-3 fatty acids upon plasma and cellular lipids, platelet function, and eicosanoid formation in humans. J Clin Invest.

[46] Bjorndal B, Strand E, Gjerde J, Bohov P, Svardal A, Diehl BW (2014). Phospholipids from herring roe improve plasma lipids and glucose tolerance in healthy, young adults. Lipids Health Dis.

[47] Yamada KA, Kanter EM, Newatia A (2000). Long-chain acylcarnitine induces Ca2+ efflux from the sarcoplasmic reticulum. J Cardiovasc Pharmacol.

[48] Bjorndal B, Brattelid T, Strand E, Vigerust NF, Svingen GF, Svardal A (2013). Fish oil and the pan-PPAR agonist tetradecylthioacetic acid affect the amino acid and carnitine metabolism in rats. PLoS One.

[49] Vigerust NF, Bjorndal B, Bohov P, Brattelid T, Svardal A, Berge RK (2012). Krill oil versus fish oil in modulation of inflammation and lipid metabolism in mice transgenic for TNF-alpha. Eur J Nutr.

[50] Ueland PM, Holm PI, Hustad S (2005). Betaine: a key modulator of one-carbon metabolism and homocysteine status. Clin Chem Lab Med.

[51] Institute of Medicine (US) Standing Committee on the Scientific Evaluation of Dietary Reference Intakes and its Panel on Folate, Other B Vitamins, and Choline. Choline. In: Book Dietary Reference Intakes: Thiamin, Riboflavin, Niacin, Vitamin B-6, Vitamin B12, Panthothenic Acid, Biotin, and Choline. Washington (National Acedemies Press); 1998. p. 390–422. 23193625

[52] Zeisel SH, Mar MH, Howe JC, Holden JM (2003). Concentrations of choline-containing compounds and betaine in common foods. J Nutr.

[53] Vennemann FB, Ioannidou S, Valsta LM, Dumas C, Ocke MC, Mensink GB, et al. Dietary intake and food sources of choline in European populations. Br J Nutrit. 2015;12:1**–**10 10.1017/S0007114515003700 26423357

[54] Gammone MA, Riccioni G, D'Orazio N (2015). Carotenoids: potential allies of cardiovascular health?. Food Nutr Res.

[55] Clayton PR, Ladi S (2015). From alga to omega; have we reached peak (fish) oil?. J R Soc Med.

[56] Strand E, Bjorndal B, Nygard O, Burri L, Berge C, Bohov P (2012). Long-term treatment with the pan-PPAR agonist tetradecylthioacetic acid or fish oil is associated with increased cardiac content of n-3 fatty acids in rat. Lipids Health Dis.

[57] Ulbricht TL, Southgate DA (1991). Coronary heart disease: seven dietary factors. Lancet.

[58] Bjorndal B, Burri L, Wergedahl H, Svardal A, Bohov P, Berge RK (2012). Dietary supplementation of herring roe and milt enhances hepatic fatty acid catabolism in female mice transgenic for hTNFalpha. Eur J Nutr.

[59] Bjorndal B, Ramsvik MS, Lindquist C, Nordrehaug JE, Bruheim I, Svardal A (2015). A phospholipid-protein complex from antarctic krill reduced plasma homocysteine levels and increased plasma trimethylamine-N-oxide (TMAO) and carnitine levels in male Wistar rats. Mar Drugs.

